# Social appearance anxiety and body checking behavior in the relationship between social media addiction and depressive symptoms among college students

**DOI:** 10.3389/fpsyt.2025.1608527

**Published:** 2025-08-20

**Authors:** Xiaochen Wang, Yanan Wang, Shufei Yang, Zhen Li, Chang Fu

**Affiliations:** ^1^ Department of Health Service and Management, School of Health and Management, Binzhou Medical University, Yantai, Shandong, China; ^2^ Department of Rehabilitation Therapy, School of Medicine, Qingdao Huanghai University, Qingdao, Shandong, China; ^3^ School of Special Education and Rehabilitation, Binzhou Medical University, Yantai, Shandong, China; ^4^ Yantai Center for Disease Control and Prevention, Yantai, Shandong, China

**Keywords:** social media addiction, depressive symptoms, social appearance anxiety, body checking behavior, college students

## Abstract

**Background:**

The relationship between social media addiction and depressive symptoms has been extensively studied; however, the underlying mechanisms remain unexplored. This study examined the mediating roles of social appearance anxiety and body checking behaviors in this relationship. This research aims to address the gap in understanding how social media addiction influences mental health, particularly among college students.

**Methods:**

Data from 1,456 students recruited from three universities in Shandong, China, were analyzed using structural equation modeling. Demographic characteristics of the participants were collected and assessed for social appearance anxiety, body checking behaviors, social media addiction, and depressive symptoms.

**Results:**

The prevalence of depressive symptoms was 13.9% in the study sample. Social appearance anxiety, body checking behavior, and social media addiction were significantly and positively correlated with depressive symptoms (*r* = 0.367 - 0.519, *p* < 0.001). Social media addiction directly and positively predicted depressive symptoms with a direct effect value of 0.173. Furthermore, social appearance anxiety and body checking behavior played independent and serial multiple mediating roles in the association between social media addiction and depressive symptoms, with indirect effect values of 0.193, 0.035, and 0.235, respectively.

**Conclusion:**

Social media addiction is a significant risk factor contributing to depressive symptoms among college students. This study provides new insight into the mechanisms underlying the relationship between social media addiction and mental health, with implications for global mental health interventions. Specifically, the negative impact of social media addiction is mediated by social appearance anxiety and body checking behavior. These findings suggest that school administrators and policymakers should develop targeted interventions to reduce social media addiction among college students and prioritize providing psychological support to alleviate social appearance anxiety and promote a healthy body image. By addressing these factors, this research contributes to a broader understanding of mental health challenges in the digital age.

## 1 Introduction

Mental health challenges among college students, particularly depressive symptoms, have become a critical public health concern worldwide (1). Previous studies have reported that the prevalence of depressive symptoms was 21.12% among Chinese college students during the COVID-19 pandemic (2) and had lasting repercussions on academic achievement and risk of self-harm (3, 4). Researchers have identified that, in addition to demographic and sociological factors such as gender and residence (5), social media use significantly influences depressive symptoms among college students in the information age (6). This paper argues that social media addiction—a behavioral pattern affecting 18.4% of students globally (7)—serves as a key modifiable risk factor for depression, mediated by maladaptive behaviors such as social appearance anxiety and body checking behavior. By examining these underexplored pathways, this study aims to bridge gaps in both theoretical understanding and culturally tailored interventions.

The dual-edged nature of social media is well documented: while it facilitates connectivity, excessive use is correlated with psychological distress through multiple mechanisms. Empirical evidence highlights that compulsive engagement disrupts sleep (8), impairs time management (9), and replaces face-to-face interactions, eroding the social support networks that are essential for resilience (10). Notably, prior research has predominantly focused on Western populations, overlooking cultural nuances in body image and self-presentation within Asian contexts. For example, platforms such as WeChat and Little Red Book emphasize visual self-curation, potentially intensifying social appearance anxiety, a predictor of depression rarely examined in this demographic. Current literature also lacks integration of behavioral mediators (e.g., body checking) despite their established link to social media-induced distress. This study addresses these gaps by contextualizing addiction–depression pathways against the background of East Asian academic and aesthetic norms.

This study investigates how social media addiction influences depressive symptoms among Chinese college students, with social appearance anxiety and body checking behavior as sequential mediators. The research makes three key contributions to the field. First, it unravels culturally specific mechanisms by testing a novel mediation model in a non-Western setting, addressing the underrepresentation of East Asian populations in existing literature. Second, it extends theoretical frameworks of the Tripartite Influence Model by incorporating behavioral responses to digital stressors, providing a more comprehensive understanding of the pathways linking social media use to depression. Third, the findings offer practical insight for designing targeted interventions to mitigate appearance-related anxiety in visually driven online environments. By employing a cross-sectional design with validated scales, this study provides timely, empirically grounded evidence for policymakers and mental health practitioners aiming to improve college students’ mental health in the digital age.

## 2 Literature review and research hypothesis

Social media provides a platform for interaction and information sharing. However, its improper use can negatively impact students’ psychological, cognitive, emotional, and behavioral well-being. Frequent social media use can directly contribute to anxiety and loneliness during social interactions as well as poor time management and increased academic stress (9). Moreover, immoderate social media use can affect sleep quality, thereby negatively impacting mental health and increasing depressive symptoms (11). Furthermore, as college students increasingly rely on social media, they may have fewer opportunities for in-person interactions, which can ultimately deteriorate their social support networks (12). This loss of social support can lead to isolation during academic stress and personal challenges, thereby increasing the risk of anxiety and depression (10). Thus, this study proposes the following hypothesis:

H1: Social media addiction is positively associated with depressive symptoms.

Social appearance anxiety is caused by individuals’ concerns regarding how other people evaluate their body image and appearance in real or imagined social situations as well as their expectations of these evaluations (13). This anxiety results from the interaction between personal psychological issues and social comparisons. The social comparison theory posits that individuals assess themselves by comparing themselves to others (14). College students often encounter meticulously curated images of other individuals’ lives and appearances on social media platforms. This constant exposure can result in negative self-comparisons, ultimately increasing social appearance anxiety (15). When students are immersed in social media, they are more likely to compare themselves to others and become preoccupied with their lives and appearances (16). This excessive focus exacerbates social appearance anxiety and potentially diminishes self-worth, which may cause depressive symptoms (17). Consequently, social media addiction can intensify depressive symptoms by exacerbating anxiety regarding social appearance. Thus, this study proposes the following hypothesis:

H2: Social appearance anxiety mediates the relationship between social media addiction and depressive symptoms.

Self-identity theory emphasizes individuals’ perceptions and feelings regarding their self-image (18). On social media, individuals are often influenced by their physical appearance and external evaluations, resulting in a strong correlation between self-identity and appearance (19). When social media addiction leads individuals to engage in frequent body checks, the realization that they may not meet societal standards can damage their self-identity. Perceived damage negatively affects psychological well-being and may cause depressive symptoms. Thus, this study proposes the following hypothesis:

H3: Body checking behavior mediates the relationship between social media addiction and depressive symptoms.

According to the sociocultural model of body image (20), frequent exposure to idealized beauty standards on social media can heighten social appearance anxiety, which is a persistent fear of negative evaluation regarding one’s physical appearance (21). This anxiety, in turn, may trigger compulsive body checking behaviors (e.g., frequent mirror checking, self-weighing), which reinforce negative self-perception and amplify emotional distress (22). Individuals with high social media engagement exhibit elevated appearance-related concerns, leading to maladaptive body monitoring and subsequent depressive symptomatology. Within the context of young adults—particularly college students—this sequential mediation may be exacerbated by developmental sensitivities to peer approval and self-image. Thus, this study proposes the following hypothesis:

H4: Social media addiction has indirect negative effects on depressive symptoms through the sequential mediating effects of social appearance anxiety and body checking behaviors.

## 3 Methods

### 3.1 Study design

This study adhered to the STROBE cross-sectional reporting guidelines to ensure transparency and completeness in reporting observational research. A multistage sampling method was employed to select participants. First, three medical universities in Shandong Province were randomly selected using convenience sampling. Subsequently, within each university, the nursing major was sampled along with one clinical medicine major and one non-clinical medicine-related major. Finally, within each selected major, several intact classes were chosen via cluster sampling, and all students in these classes were surveyed. From September to November 2024, a total of 1,694 electronic surveys were distributed through the Questionnaire Star platform.

The participants were selected based on the following inclusion criteria: (1) having regular access to the internet, (2) being able to read and write in Chinese, and (3) providing voluntary consent to participate in the study. Exclusion criteria included incomplete responses or logical inconsistencies in responses (23). The sample size for this study was determined based on the principle that the number of participants should be 10–15 times the total number of items in the survey. Given that the survey contained 62 items, a preliminary sample size of 62 × 15 = 930 participants was calculated. To account for potential incomplete or inconsistent responses, the total number of questionnaires distributed was increased to 1,694. After excluding invalid responses, the final valid sample consisted of 1,456 participants, resulting in a valid response rate of 86.4%. This sample size was deemed sufficient to ensure the robustness and reliability of the statistical analyses.

To ensure data quality and avoid duplication or fraud, the following strategies were implemented: (1) unique identifiers were used to track participants and prevent duplicate submissions, and (2) responses with identical answers or unusually short completion times were flagged and excluded. Ethical approval for this study was obtained from the Ethics Committee of Binzhou Medical University. Participants provided written informed consent before completing the survey. Participation was voluntary, and no incentives were offered to ensure the integrity of the data. All data were anonymized and stored securely to protect participants’ privacy. The data will be shared upon reasonable request, in accordance with the ethical guidelines of the institution and relevant regulations.

### 3.2 Measurements

#### 3.2.1 Social media addiction

The Bergen Social Media Addiction Scale assessed social media addiction (24), which evaluates the frequency of negative effects of social media use on participants’ lives over the past year. This scale has been extensively used among Chinese college students and exhibited excellent reliability and validity (25). It consists of six items, such as “How often during the last year have you spent a lot of time thinking about social media or planned use of social media?” and “How often during the last year have you felt an urge to use social media more and more?” Each item is rated on a five-point Likert scale ranging from 1 (*very rarely*) to 5 *very often*), yielding a total score that varies between 6 and 30. Higher scores indicate more severe social media addiction (26). The scale’s Cronbach’s α coefficient was 0.898 in this study.

#### 3.2.2 Social appearance anxiety

The Social Appearance Anxiety Scale measured social appearance anxiety (27). The Chinese version of this scale has been widely used to examine the overall level of social appearance anxiety among college students (21). This scale comprises 16 items designed to assess anxiety regarding negative evaluations by others based on overall appearance (including body image). These items include statements such as “I am concerned people will find me unappealing because of my appearance” and “I feel anxious when other people say something regarding my appearance.” Each item on this scale is rated from 1 (*not at all*) to 5 (*extremely*), resulting in total scores ranging from 16 to 80. Higher scores indicate greater levels of social appearance anxiety (28). The scale’s Cronbach’s α coefficient was 0.962 in this study.

#### 3.2.3 Body checking behavior

The Body Checking Questionnaire was used to assess body checking behaviors among college students (29). The scale comprises 23 items, which are organized into three aspects: (1) overall appearance checking, illustrated by the item “I check my reflection in glass doors or car windows to see how I look”; (2) specific body part checking, exemplified by “I check the diameter of my legs to make sure they’re the same size as before”; and (3) special checking, such as “I compare myself to models on TV or in magazines.” Each item is rated on a five-point Likert scale ranging from 1 (*never*) to 5 (*always*), with total scores ranging from 23 to 115. Higher scores indicate stronger feelings of body dissatisfaction (30). The scale’s Cronbach’s α coefficient was 0.968 in this study.

#### 3.2.4 Depressive symptoms

Depressive symptoms were assessed using the Patient Health Questionnaire-9 (PHQ-9) (31). The total score of the PHQ-9 ranges from 0 to 27, with the scores of its nine items ranging from zero (not at all) to three (almost daily). For example, participants were asked questions such as “Over the last two weeks, how often have you been bothered by little interest or pleasure in doing things?” and “Over the last two weeks, how often have you been bothered by feeling down, depressed, or hopeless?” A cutoff score of 10 was used, with scores below and higher than 10 indicating no and presence of depressive symptoms, respectively (32). The scale’s Cronbach’s α coefficient was 0.881 in this study.

### 3.3 Statistical analyses

Data analysis was performed using SPSS version 26.0. Continuous variables were described using means ± standard deviations, whereas categorical variables were expressed as percentages. Cronbach’s α coefficient assessed scale reliability. Pearson’s correlation coefficients analyzed the relationships between variables. The P-P plots indicated that the dependent variable (depressive symptoms) was normally distributed. A chain mediation model was developed using AMOS 24.0, with social media addiction (X) as the independent variable, depressive symptoms (Y) as the dependent variable, and social appearance anxiety (M1) and body checking behavior (M2) as the two sequential mediators. The model was specified and estimated directly within AMOS, and a custom model code was implemented to calculate the direct, indirect, and total effects of the mediators. To test the significance of the indirect effects, a non-parametric bootstrap method with 5,000 resamples was employed. Additionally, bootstrapping provides bias-corrected confidence intervals, which are more robust than traditional methods for assessing indirect effects. By conducting the analysis directly within AMOS and employing the bootstrap method, the study ensured a robust and flexible approach to testing the mediation hypotheses, particularly given the complexity of the chain mediation model.

## 4 Results

### 4.1 Common method bias testing

To assess common method bias, we utilized the Harman single-factor method. The analysis revealed that six factors had eigenvalues greater than 1, with the factor accounting for the highest variance explaining 25.18% of the total variance. Because this percentage is below the 40% threshold, it indicates the absence of significant common method bias in our study.

### 4.2 Descriptive statistics

The sociodemographic characteristics of the 1,456 participating college students indicated that 67.7% of female participants had a mean age of 19.49 ± 1.33 years. More than half were from urban areas (51.2%), and the majority were single (76.1%). Non-clinical majors and second-year students accounted for 58.4% and 34.1% of the sample, respectively. Depressive symptoms were reported by 13.9% of the participants. Participants with a total score of 19 or higher on the social media addiction scale accounted for 15.9% of the sample. The average value and standard deviations for social media addiction, social appearance anxiety, and body checking behavior were 14.41 ± 5.32, 34.88 ± 14.02, and 41.13 ± 18.34, respectively. Demographic details are presented in **Table 1**.

**Table 1:** Sociodemographic characteristics of study participants.

Variables	N(%)
Gender	
Male	471(32.3)
Female	985(67.7)
Age, (Mean ± SD)	19.49 ± 1.33
Residence	
Urban	746(51.2)
Rural	710(48.8)
Relationship status	
Single	1107(76.1)
Not single	349(23.9)
Major categories	
Nursing or clinical medicine	606(41.6)
Non-clinical medicine	850(58.4)
Grade	
Freshman	490(33.7)
Sophomore	497(34.1)
Junior	366(25.1)
Senior and above	103(7.1)
Depressive symptoms	
<10	1253(86.1)
≥10	203(13.9)
Social media addiction	
<19	1224(84.1)
≥19	232(15.9)
Depressive symptoms,(mean ± SD)	5.41 ± 4.49
Social media addiction,(mean ± SD)	14.41 ± 5.32
Social appearance anxiety,(mean ± SD)	34.88 ± 14.02
Body checking behavior,(mean ± SD)	41.13 ± 18.34

SD, standard deviation.

### 4.3 Correlation analysis

The correlation analysis results indicated significant positive correlations among all four variables (**Table 2**).

**Table 2:** Pearson’s correlation coefficients between depressive symptoms and social media addiction, social appearance anxiety, body checking behavior among college students in China.

Variable	Social media addiction	Social appearance anxiety	Body checking behavior	Depressive symptoms
Social media addiction	1			
Social appearance anxiety	0.421^***^	1		
Body checking behavior	0.471^***^	0.576^***^	1	
Depressive symptoms	0.367^***^	0.519^***^	0.405^***^	1

^***^ *p* < 0.001

### 4.4 Structural model and bootstrap test

The model fit indices were satisfactory: CMIN/DF = 4.732 < 5, GFI = 0.838, AGFI = 0.823, TLI = 0.917, and CFI = 0.922, all of which exceed 0.8, whereas RMSEA = 0.051 < 0.08. Based on previous studies, factors such as gender, age, major, relationship status, residence, grade, and monthly expenses have been identified as influential factors for depressive symptoms (33, 34). Therefore, in our mediation analysis, we incorporated these variables as covariates to control for their potential confounding effects and to more accurately examine the relationships among the main variables of interest.

**Figure 1:**
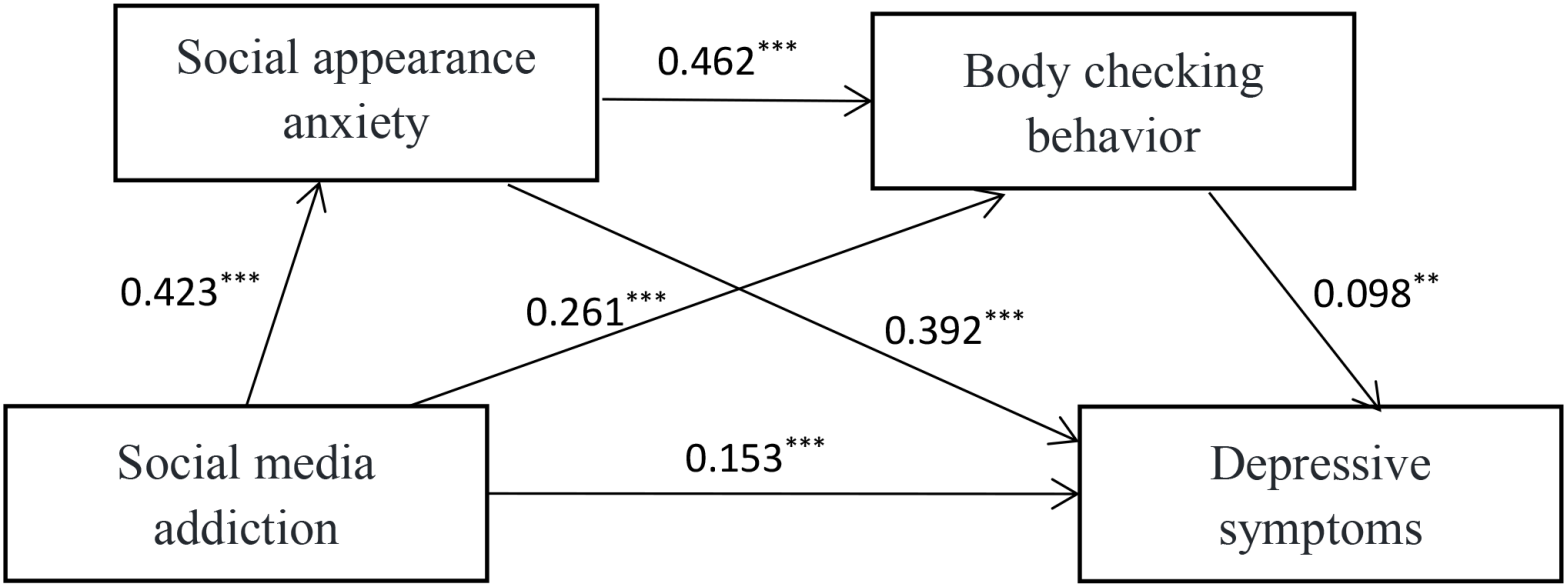
The multiple mediation of social appearance anxiety and body checking behavior between social media addiction and depressive symptoms. ^**^p < 0.01, ^***^p < 0.001.

As shown in **Figure 1**; **Table 3**, the mediation analysis demonstrated that social media addiction exerted a significant positive relationship with social appearance anxiety (β = 0.423, p < 0.001). When both social media addiction and social appearance anxiety were included as predictors, each significantly predicted levels of body checking behavior (β = 0.261, p < 0.001 and β = 0.462, p < 0.001, respectively). In a model incorporating social media addiction, social appearance anxiety, and body checking behavior as predictors, social media addiction exhibited a significant positive relationship with depressive symptoms (β = 0.153, p < 0.001), and social appearance anxiety had a significant positive relationship with depressive symptoms (β = 0.392, p < 0.001). Similarly, body checking behavior demonstrated a significant positive relationship with depressive symptoms (β = 0.098, p < 0.01).

**Table 3:** Regression analysis between variables.

Regression equation		Overall fit indices			Significance of regression coefficient	
Outcome variable	Predictor variable	R	R^2^	F	β	t
Social media addiction	Depressive symptoms	0.389	0.152	32.335^***^	0.363	14.831^***^
Social appearance anxiety	Social media addiction	0.431	0.186	41.315^***^	0.423	17.623^***^
Body checking behavior	Social media addiction	0.649	0.421	116.927^***^	0.261	11.708^***^
	Social appearance anxiety				0.462	20.846^***^
Depressive symptoms	Social media addiction	0.559	0.313	65.830^***^	0.153	6.002^***^
	Social appearance anxiety				0.392	14.223^***^
	Body checking behavior				0.098	3.427^**^

**p < 0.01, ***p < 0.001. Gender, age, major, relationship status, residence, grade and monthly expense were done as control variables included in the model. The study variables were standardized.

Using a bias-corrected percentile bootstrap, the study sample was resampled 5,000 times to construct a 95% confidence interval (CI) and test the mediating effects of social appearance anxiety and body checking behaviors on the relationship between social media addiction and depressive symptoms. The results of the multiple mediation analysis of social appearance anxiety and body checking behavior in the relationship between social media addiction and depressive symptoms based on the bootstrap method are shown in **Table 4**. The total indirect effect of social media addiction through social appearance anxiety and body checking behavior on depressive symptoms was statistically significant (estimate = 0.252, 95% CI [0.209, 0.300]). The single mediation effects of social appearance anxiety (estimate = 0.193, 95% CI [0.156, 0.236]) and body checking behavior (estimate = 0.035, 95% CI [0.009, 0.061]) were statistically significant. Additionally, the serial mediation effect of social appearance anxiety and body checking behavior (estimate = 0.025, 95% CI [0.006, 0.045]) was significant.

**Table 4:** Standardization effect and direct effect in the model.

Path	Standardized estimate	*p*	95% confidence interval		Ratio of effect
			Lower	Upper	
Social media addiction→ Social appearance anxiety→ Depressive symptoms	0.193	< 0.001	0.156	0.236	42.70%
Social media addiction→Body checking behavior→ Depressive symptoms	0.035	0.007	0.009	0.061	7.74%
Social media addiction→ Social appearance anxiety→ Body checking behavior→ Depressive symptoms	0.025	0.008	0.006	0.045	9.96%
Total Indirect effect	0.252	< 0.001	0.209	0.300	59.29%
Direct effect	0.173	< 0.001	0.094	0.247	40.71%
Total effect	0.425	< 0.001	0.361	0.486	

## 5 Discussion

To our knowledge, this is the first study to explore the serial multiple mediation of social appearance anxiety and body checking behavior in the relationship between social media addiction and depressive symptoms among college students in China. The survey results revealed a depressive symptoms rate of 13.9% among participating college students. This rate, when compared to existing literature, was higher than the 11.6% reported in Japan but lower than the 21.12% observed during the COVID-19 pandemic in China (2, 35). The observed variability in depression rates may be partially attributed to the intense academic demands of the medical college from which the data were derived (36). Moreover, the significant psychological impact of the COVID-19 pandemic on students may have led to increased awareness and coping strategies, potentially resulting in subsequent alleviation of mental health issues during the post-pandemic period (37). This study found a 15.9% prevalence rate of social media addiction, determined based on a cutoff score of 19 (38). When compared to previous studies reporting rates ranging from 10.2% to 30.11% (39, 40), although it falls within the moderate range, educational administrators should still pay close attention to this finding. The variability in reported social media addiction rates could be due to differences in study populations, measurement tools, and cultural contexts across various studies.

Furthermore, this study revealed a positive correlation between social media addiction and depressive symptoms among college students, which was consistent with the findings of a previous study (41). As supported by existing literature, as social media usage increases, students may experience increased loneliness and anxiety and diminished self-worth, potentially exacerbating depressive symptoms (42). Moreover, immoderate social media use can disrupt normal sleep patterns and daily routines, leading to sleep deprivation and reduced quality of life and further contributing to depressive tendencies (8). To effectively mitigate the negative effects of social media addiction, students should cultivate healthy social media habits to alleviate depressive symptoms and promote their overall mental well-being.

Social appearance anxiety mediated the relationship between social media addiction and depressive symptoms in college students in this study, which is consistent with a previous study (17). The abundance of idealized images and lifestyles on social media platforms may lead college students to engage in unfavorable self-comparisons and exacerbate their dissatisfaction with their appearance (43). Perceiving a significant gap between oneself and these idealized standards can cause appearance anxiety (44). Moreover, appearance anxiety can cause discomfort in social situations, further intensifying loneliness and depression among students (13), which aligns with existing knowledge on the psychological impact of appearance anxiety.

This study found that body checking behavior mediates the relationship between social media addiction and depressive symptoms among college students. One explanation for this phenomenon, in line with the literature, is that the prevalence of idealized appearance standards on social media platforms may lead to increased self-focus and body checking behaviors. Repetitive self-scrutiny (such as body checking behaviors) can induce anxiety and stress, potentially elevating the risk of depressive symptoms (35). Another explanation is that body checking behavior was positively associated with eating disorders (45), which can lead to depressive symptoms (46), as previously reported in research on the connection between body-related behaviors and mental health.

The data showed that social appearance anxiety was associated with body checking behaviors among college students. As described in relevant literature, driven by social appearance anxiety, college students may become more attentive to the details of their appearance, such as skin condition, body proportions, and dress code. They may exhibit more frequent body checking behavior (such as looking in the mirror as well as seeking evaluations and feedback from others (29)), which is in line with the understanding of how appearance-related anxiety influences body-checking actions.

This study had several theoretical and methodological limitations that warrant in-depth consideration. First, it was impossible to determined causality because this was a cross-sectional study. Although structural equation modeling analysis provided statistical indications of the associations between body checking behavior, depressive symptoms, social media addiction, and appearance anxiety, longitudinal studies are necessary to elucidate potential causal relationships and interaction mechanisms among these variables. This is a significant theoretical shortcoming as current theories often assume a certain causal pattern, but our cross-sectional design fails to confirm one. Second, the use of self-report questionnaires for data collection may have been subject to recall bias. This is a methodological flaw as self-reported data can be influenced by various factors such as social desirability, leading to inaccurate information. Third, the use of convenience sampling exclusively from medical colleges severely restricts the generalizability of our findings to the broader population of higher education students or other demographic groups. This not only limits the practical application of our results but also challenges the theoretical scope of our study. Additionally, the study did not assess potential non-response bias or differences between early and late responders. Although the valid response rate was high (86.4%), non-respondents might systematically differ from participants in ways that could influence the results (e.g., lower motivation or higher stigma related to body image concerns). Similarly, early responders (who may be more engaged or affected by the topic) might differ from late responders (who may participate due to reminders or incentives), potentially introducing response timing bias. Future research could employ follow-up analyses (e.g., comparing demographic/clinical characteristics of early vs. late respondents) to evaluate these biases.

In summary, this study contributes to the understanding of the mechanisms underlying the relationship between social media addiction and depressive symptoms among college students. By identifying social appearance anxiety and body checking behavior as serial mediators, the findings extend existing theories of social media’s psychological impact. The results highlight the importance of appearance-related concerns and self-focused behaviors as critical pathways to depressive symptoms, offering potential avenues for future theoretical development and intervention strategies.

In terms of future research, longitudinal studies could be conducted to establish the causal relationships and temporal dynamics among social media addiction, social appearance anxiety, body checking behavior, and depressive symptoms. Additionally, experimental designs could be employed to test the causal role of these mediators in the development of depressive symptoms, providing stronger evidence for their theoretical significance. To address the methodological limitation of self-report questionnaires, future studies could use multiple data collection methods, such as objective social media usage tracking and clinical psychological assessments. To improve the generalizability of the findings, future research should expand the sample size to include students from different regions, majors, and cultural backgrounds.

## 6 Implications

Based on these findings, we proposed several recommendations for reducing depressive symptoms among college students. First, from a practical perspective, college administrators should prioritize addressing depressive symptoms among students. Institutions should organize mental health awareness campaigns to enhance students’ understanding of the importance of psychological well-being. These initiatives should focus on alleviating academic demands and promoting mental health maintenance. For example, interventions based on cognitive-behavioral therapy could be designed to help individuals reduce excessive social media use and improve their body image perception, thereby alleviating depressive symptoms. In practice, colleges can set up dedicated mental health rooms in which students can seek immediate support when they feel depressed. Moreover, they should establish a peer-support system through which students with positive mental states can communicate with and help those with depressive symptoms. Second, in the field of education, college administrators should provide relevant health education to college students to help them develop healthy social media use habits. Schools and communities could also initiate educational programs, such as courses, lectures, workshops, and seminars, to help adolescents and young adults recognize the relationship between social media use and mental health and encourage them to adopt healthier coping strategies. Teachers can incorporate case studies related to social media addiction and body image anxiety into their teaching, guiding students to consider how to maintain good mental health in the digital age. Finally, from a management perspective, college administrators should actively implement psychological health education and counseling programs that target social appearance anxiety. These initiatives should aim to help students develop healthy self-perceptions and reduce excessive and frequent body checks as well as preoccupation with physical appearance. Colleges can formulate strict management regulations on social media use during study hours to reduce the interference of social media on students’ learning and mental health. At the same time, they can allocate sufficient funds for mental health counseling services to ensure the quality and effectiveness of these services.

In summary, the study findings provide a solid theoretical and practical foundation for future psychological interventions. By addressing social media addiction, body image anxiety, and depressive symptoms, these interventions can lead to more effective mental health outcomes.

## 7 Conclusion

This study underscores the significant prevalence of social media addiction and depressive symptoms among Chinese college students. Critically, the findings demonstrate that social media addiction contributes directly to depressive symptoms and, more significantly, exerts its influence indirectly through a sequential psychological pathway: first amplifying social appearance anxiety and subsequently increasing engagement in body checking behaviors. This establishes body checking behaviors as a core mechanism linking digital overuse to negative affect. Consequently, addressing depressive symptoms in this population requires targeted interventions focused specifically on mitigating social media addiction, alleviating social appearance anxiety, and reducing the frequency and impact of maladaptive body checking behaviors. These findings provide a crucial theoretical and empirical foundation for developing effective strategies to enhance student mental health.

## Data Availability

The raw data supporting the conclusions of this article will be made available by the authors, without undue reservation.
